# Resident Physicians’ Perceptions of Artificial Intelligence and Implications for Medical Education: A Qualitative Study

**DOI:** 10.12688/mep.20833.1

**Published:** 2025-07-02

**Authors:** Andrew McFarlane, Shirin Sadri, Ezra Schwartz, Deepthiman Gowda

**Affiliations:** 1 Case Western Reserve University School of Medicine, Cleveland, Ohio, USA; 2 Stanford University School of Medicine, Stanford, California, USA; 3 McGill University Faculty of Medicine, Montreal, Québec, Canada; 4 Kaiser Permanente Bernard J Tyson School of Medicine, Pasadena, California, USA

**Keywords:** Artificial Intelligence, Medical Education, Physician Perspective

## Abstract

**Background:**

Educators have called for training in artificial intelligence (AI) in medical education given its certain impact on the future of healthcare. However, there is no consensus regarding how to introduce AI into medical education and little is known about how AI is viewed among medical trainees. In an effort to inform the development of medical education curricula on AI, this study explores perceptions of resident physicians regarding AI in healthcare and its possible impact on their future practice.

**Methods:**

The authors conducted focus groups with resident physicians across multiple specialties in 2018–2019. Residents were invited to voluntarily participate during pre-existing conference times. Interview transcripts were coded iteratively, and coded data was clustered into categories and themes to capture resident perceptions on AI.

**Results:**

Fifty-six residents from emergency medicine, internal medicine, pathology, pediatrics, and radiology participated in six separate focus groups. Conversations generated the following five overarching themes: healthcare is transforming, AI has a role at the clinical and systems level, concern for lack of agency in the development and implementation of AI, AI presents potential harms and uncertainties, and enduring roles of the physician: humanism, judgment, and responsibility.

**Conclusion:**

Residents described humanistic roles that should not be replaced by technology and voiced concerns that physicians lack agency to influence how AI will be used in healthcare. Medical education should explore humanistic and ethical challenges related to AI, provide a foundational understanding of AI technology, and offer opportunities for participation in the development of AI technology when possible.

## Introduction

The recognition that AI will deeply impact the future of healthcare is widely accepted, though the range of narratives around the potential uses, benefits, and risks to clinical practice vary ^1– 6^. As a result of the impending use of AI in healthcare, there have been calls to incorporate training in AI into medical education ^7, 8^. Despite these calls, recent reviews of the literature noted a lack of consensus regarding how to introduce AI into medical education ^9, 10^. In keeping with approaches for curricular development described by Thomas *et al*., ^11^ a better understanding of trainees’ needs and perspectives regarding AI is needed as educators begin to develop curricula in this area.

Research on perceptions of AI amongst practicing physicians found hope for improved clinical efficiency along with a concern for possible loss of physician jobs ^12^. However, little is known about the perceptions of trainees regarding AI, including resident physicians who are on the threshold of entering practices increasingly influenced by AI. Residents’ perceptions of AI may be different from those of physicians already in practice, given that the current generation of trainees have been educated in an era of ubiquitous digital technology. Also, residents manifest the knowledge, mindset, and skills that result from current medical education curricula, thus offering a unique insight into the current state of medical education.

 In an effort to inform the development of medical education on AI, this paper reports the findings of a qualitative study exploring the perceptions of AI among resident physicians across multiple specialties.

## Methods

### Study approach

A constructivist grounded theory (CGT) approach, as described by Charmaz ^13^, was applied to the topic of interest. Focus group discussions were conducted in order to leverage interaction between participants, and thus enabled the exploration of individual and group level perceptions regarding AI.

### Participants and context

Resident physicians at New York-Presbyterian Hospital-Columbia University Irving Medical Center (NYP-CUIMC) in New York, NY were invited to participate in this study. Initial participant recruitment included residents from dermatology, internal medicine, pathology, and radiology; these specialties were chosen as initial participants given documented robust application of AI in these fields ^14– 17^. Given emerging focus group conversations that included the impact of AI more broadly in healthcare, an iterative sampling strategy (in keeping with methods of CGT) resulted in an expansion of sampling to include focus groups with residents in emergency medicine and pediatrics. Because the research interest was less focused on procedural applications of AI, surgical specialties were not recruited. Dermatology did not respond to focus group requests.

A representative from each specialty of interest (e.g., faculty member or chief resident) was identified as a liaison to assist with participant recruitment. Representatives invited residents across all postgraduate levels to participate via email, verbal announcements at meetings, and informal word-of-mouth.

Written informed consent was obtained from each participant. Participation was voluntary and did not affect resident standing, and no compensation or incentive was provided to participants. Participants were informed that focus group data would be kept confidential and would not be shared with residency leadership.

This study was approved by the Institutional Review Board of CUIMC (#AAAR7376; 4/12/2018).

### Data collection

An initial focus group guide was developed around the primary research question. Prior literature on perceptions of AI was used to inform this initial guide. In keeping with a CGT approach, the research team gathered regularly throughout the data collection period to code data, analyze data, and iteratively update the focus group guide to explore emerging themes in subsequent focus groups ^18^.

Six specialty-specific focus group interviews were conducted between May 2018 and June 2019. Focus groups took place in private departmental conference rooms during existing, hour-long didactic or conference time, and consisted only of residents and research team members. Every focus group interview was conducted primarily by investigator A.M. The most current iteration of the focus group guide at the time of each focus group meeting was used to facilitate discussion; however, conversation during the focus groups was encouraged to flow naturally and could deviate from questions contained in the guide. Prior to initiation of the focus group, individual data was collected on level of clinical training and any prior experience with AI. Focus groups were audio recorded, transcribed by the research team, and maintained securely ^19^. All data were de-identified prior to analysis.

### Analysis

All co-authors met regularly between May 2018 and March 2020 in person and virtually to review transcripts, define emerging codes, and apply existing codes to statements across transcripts. As described by Saldaña ^20^, codes were applied to transcript text that was felt to be ‘summative, salient, essence-capturing, and/or evocative’; coded excerpts ranged from sentence to paragraph in length, and could contain single or multiple speakers. The transcript data and codes were managed securely on Dedoose ( Dedoose, Manhattan Beach, CA, USA). Saturation, when ‘no new information seems to emerge during coding’ ^21^, was achieved with analysis of the transcript from the 6th focus group, at which point additional participant recruitment ceased. Co-authors A.M. and E.S. returned to all prior transcripts to apply any newer codes and ensure consistent use of codes.

All co-authors then met virtually to identify categories evident in the codes and organized these into five themes representative of the data: healthcare is transforming, AI has a role at the clinical and systems level, concern for lack of agency in the development and implementation of AI, AI presents potential harms and uncertainties, and enduring roles of the physician: humanism, judgment, and responsibility.

### Reflexivity

The research team included D.G. and A.M., who are active clinicians. A.M. was a resident physician at the time of study initiation and is now an academic hospitalist physician. E.S. is a medical student. S.S. was a medical student at the time of the study and is now a resident physician. D.G. and E.S. have formal training and experience with using narrative medicine for purposes of professional identity formation. D.G. has extensive experience in clinical skills education and medical education leadership.

## Results

Fifty-six residents, ranging from PGY-1 to PGY-4, participated in six distinct focus groups. Prior experience with AI was most commonly reported amongst radiology residents ( Table 1); however, the majority of residents, including those within radiology, denied having experience with AI.

**Table 1.** Number of participants in focus groups of resident physicians assessing their perceptions towards artificial intelligence (AI), along with frequency of self-reported experience with AI, conducted from 2018 to 2019 at New York Presbyterian-Columbia University Medical Center.

Focus Group ^a^	Number of Participants	Number Self-Reporting Experience with AI ^b^ (%)
Radiology 1	7	3 (43%)
Radiology 2	17	7 (41%)
Pathology	9	2 (22%)
Internal Medicine	11	1 (9%)
Pediatrics	8	1 (13%)
Emergency Medicine	4	0 (0%)

^a^Focus groups were conducted separately with each specialty; two separate Radiology focus groups were held given the number of participants ^b^Self-reported to the written prompt: “Experience with Artificial Intelligence?”

The codes most frequently applied for each theme were present across the majority of the focus group transcripts. Codes for the theme regarding agency and development of AI were primarily applied to the pathology and radiology groups.

### Healthcare is transforming with AI

Residents across focus groups acknowledged that healthcare and the practice of medicine will be different in the future, and that AI will be a part of that future. Residents did not report concerns about the loss of physician jobs; rather, many residents anticipated being able to adapt alongside evolving technology.

I'm not worried about radiology because I feel like it's always been on the cutting edge of technology and medicine … I think it will look different, definitely, but I don't think that it's going to disappear. I think that we’ll just continue to change and evolve. Like we always did. - Radiology Group 2

The inevitability of AI’s presence in healthcare, and the importance of integrating it into practice, was also described.

It seems like it's [AI] just going to get more and more important and I feel like the more we can integrate, it will make everything easier for everybody. The diagnoses will be better, probably. They should be. That's why we're doing this. - Pathology

### AI has a role at the clinical and systems level

Residents across all specialties spoke about potential applications of AI, at both the clinical and systems level. Many noted that AI could assist the work of physicians, completing laborious and relatively simple tasks, thereby freeing them up to engage with more complex and/or interesting work.

There are things within radiology that are just such high volume. Like, we get about 350 chest x-rays every morning … If artificial intelligence could help us get through some of those, it would give us more time to do the things like … actually do interpretation as opposed to just sheer getting through volumes. It's like getting a bulldozer to dig a ditch versus being the architect of a building. - Radiology Group 1

Residents also spoke of the potential value of AI in leveraging certain capacities that are beyond human ability, such as advanced pattern recognition, high resolution detection, and computational power.

I think the most interesting aspect is actually not trying to replace or substitute what we already do, but to discover new things that you can provide in addition to what would otherwise be too cost inefficient for a human to do. For example, I'm not going to go around and measure every single lung nodule in the lung … But if a computer can measure every single one and it demonstrates that it can calculate a much more accurate measurement of how well the lung cancer is responding to treatment, then that would add to our practice. - Radiology Group 1

### Concern for lack of agency in the development and implementation of AI

Residents explored the question of who is in control of the development of AI technology and considered which stakeholders determine its implementation and regulation. Some residents expressed that, due to financial barriers to entry, only a few wealthy private companies or academic centers could afford to develop AI (Pathology, Radiology Groups 1 and 2, Internal Medicine). They expressed a concern that practicing physicians seemed to lack agency in the development of AI tools that would ultimately affect their work. A resident noted:

Most innovation comes from companies trying to profit. Academic institutions have a lot of money. They can't profit but they profit in prestige and other ways … it's either you have a lot of money to spend or there's a lot of money to be made, but there's not a bunch of doctors with good ideas like, ‘let's just get together and fund our own data algorithm research.’ That doesn't happen. - Pathology

Residents framed the implementation of AI in healthcare as a societal concern and not solely a medical one, and as such, generated discussions around regulatory, ethical, and political issues.

They're allowing semi-autonomous cars already? I didn’t vote for this. They are crashing into people and … yeah, of course they are. So, I feel like this is the type of thing in medicine where you have to go slow with this stuff, especially when people's lives are involved. - Radiology Group 1

Two radiology residents offered a seemingly disenfranchised perspective on the development and implementation of AI technology, stating that, ‘we’re not part of the conversation’, and that they will have to ‘roll with the punches’ (Radiology Group 2).

### AI presents potential harms and uncertainties

Residents noted several possible harms resulting from the integration of AI into medicine. Some residents identified profit generation as the driver behind AI’s development, and feared that operating inside an increasingly efficient but profit-driven system may paradoxically increase physician workload and resource utilization. (Pathology, Radiology Groups 1 and 2)

We're probably going to have to work more somehow. Like, ‘Ooh yeah, … I can read twice as many CT’s.’ That’s the MBA model. - Radiology Group 1

While many residents acknowledged and welcomed AI’s clinical benefits, one resident wondered whether increased dependence on AI in practice could result in a diminishment of one’s clinical skills.

[AI] can be helpful, but if you rely on it too much without being a doctor and thinking for yourself, there's danger to that. - Internal Medicine

Lastly, the black box phenomenon *,* referring to a lack of understanding of, and an inability to inquire into, the inner workings of the AI decision-making process, was a concern identified in multiple focus groups. (Medicine, Pathology, Radiology Groups 1 and 2).

Here's a machine that tells you ‘two plus two is four’ and you go, ‘okay, I have a question about it.’ How do you go and have that conversation? It just stops right there. - Radiology Group 2

### Enduring roles of the physician: humanism, judgment, and responsibility

Residents in all groups mentioned relationally and emotionally situated activities, such as communicating empathically with patients, explaining a diagnosis, and discussing bad news, as being critical to a physician’s role that should not be replaced by AI.

Just like difficult conversations, the human-to-human interaction … knowing that someone else is a human being and they can understand, potentially, or have some empathy for whatever the situation may be. - Pediatrics

One resident offered observation for emotion and understanding as an example of a physician’s capacity that cannot be replaced by AI:

Like looking at a parent's face and telling whether or not they’re understanding of what level you're speaking at and going either higher or lower just based on general facial expressions, without someone saying, ‘I don't understand this’. - Pediatrics

Residents also identified the ability to apply judgment and appreciate clinical context as a strength that humans uniquely bring to the care of patients.

In some aspects of medicine, what you do next depends a lot on pretty clear things … But then there's also a lot of judgment calls, and I don't think AI is close to replacing that. You have to be able to look at the patient and get a picture, because our numbers don't say everything. - Emergency Medicine
The hard part of medicine is being able to explain your findings, explain what it means, and why that matters, and what it means in the context of the patient. *-* Radiology Group 1

Residents in all fields discussed the need for humans to verify the results or decision-making of AI tools, and many reported that ultimate decision-making will still be the responsibility of physicians, as explained in the following:

Thinking about internal medicine, you'd never have a [basic metabolic panel] come out and the potassium is high and then not have a doctor be involved in this. So, I think there's always going to be some [physician] being an intermediary between what the actual findings are and then what we do with them. - Radiology Group 1

## Discussion

**Figure 1.**
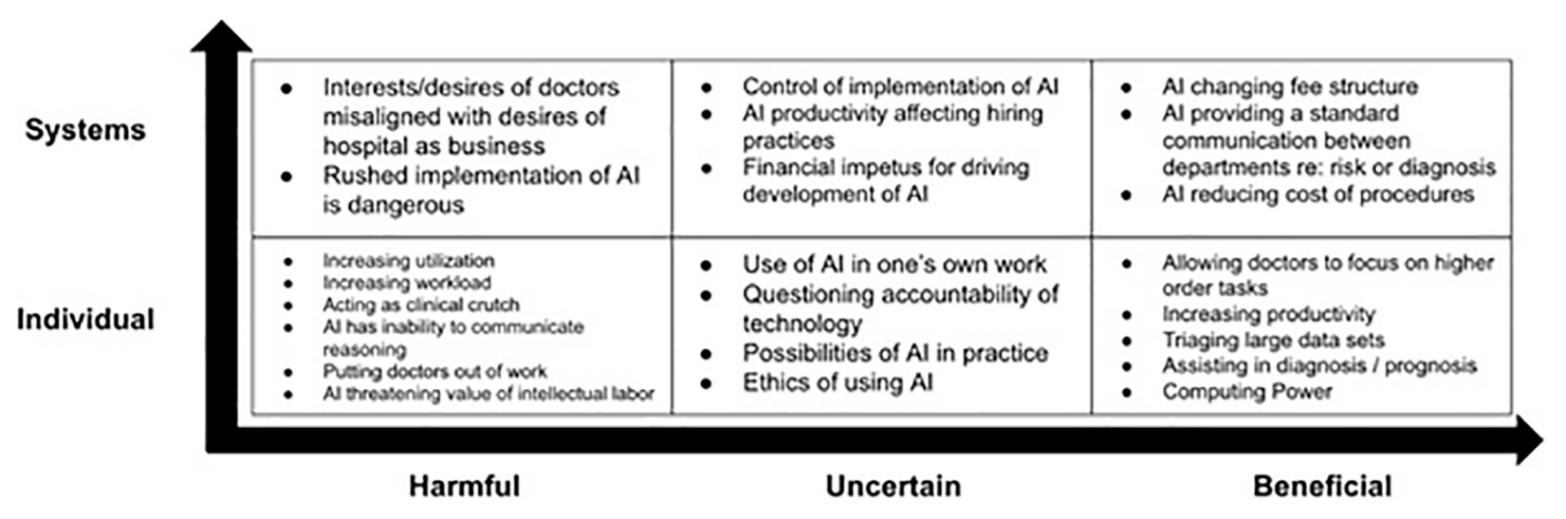
Prominent categories of perceptions towards artificial intelligence (AI) derived from focus groups of resident physicians conducted from 2018 to 2019 at New York Presbyterian-Columbia University Medical Center, organized as harmful, uncertain, or beneficial, as well as individual or systemic scope.

This study explored resident physicians’ perceptions of AI and their future clinical practice. The residents, drawn from several specialties, conveyed confidence that AI would impact the future of healthcare. They were largely optimistic about AI’s potential to improve healthcare as well as their ability to adapt alongside new technologies, but also identified some concerns and potential harms of AI. The prominent findings from the focus groups have been organized in Figure 1 in terms of beneficial or harmful effects and individual or systemic scope.

Consistent with prior research, this study revealed a flexibility in residents’ perceptions of their roles in relation to the emergence of technology ^22, 23^. Despite this openness, however, residents noted several critical humanistic duties of physicians that should not be relegated to AI. Residents also noted that if AI creates efficiencies in patient care, physicians may have time freed up to attend to those humanistic duties. Those person-centered, humanistic duties such as empathically receiving the patient story, emotionally supporting patients, and educating patients should continue to be emphasized in the medical curriculum, and may become even more critical to the physician role with the advancement of technology. Narrative medicine and the humanities are essential to promoting the development of these skills ^24, 25^, and should be required, formal components of medical education. Additionally, discussions on the boundaries separating tasks appropriate for AI versus those performed by physicians should be explored in doctoring and medical ethics curricula. The implications for medical education curriculum development based on each of the five themes identified from the focus groups can be found in Table 2.

**Table 2.** Implications for medical education of each of the five themes identified from focus groups of resident physicians assessing their perceptions towards artificial intelligence (AI) Conducted from 2018 to 2019 at New York Presbyterian-Columbia University Medical Center.

Themes	Implications for Medical Education
Healthcare is transforming with AI	● Introduce foundational AI concepts into formal curricula. ● Inform students of the use of AI in their current clinical training environments.
AI has a role at the clinical and systems level	● Explore the use of AI to support clinical practice. ● Explore the use of AI at a systems level to improve the health of populations.
Concern for lack of agency in development and implementation of AI	● Educate students on the conceptual and technical foundations of AI. ● Provide elective opportunities to deepen knowledge and experience in AI. ● Facilitate opportunities for student involvement in interdisciplinary AI research.
AI presents potential harms and uncertainties	● Ensure students’ clinical decision-making is supported by an understanding of pertinent biomedical sciences. This should remain the basis for practice even as clinical settings are increasingly influenced by AI. ● Include training on how to critically appraise and integrate AI tools into clinical decision-making.
Enduring roles of the physician: humanism, judgment, and responsibility	● Humanism ○ Incorporate the health humanities and narrative medicine into curricula to develop skills of attention and better understand the patient’s story and context of care. ○ Emphasize history-taking and physical examination as enduring and foundational skills of clinical care. Careful listening and clinical touch are irreplaceable tools for information gathering and empathic connection. ● Clinical Judgment ○ Reinforce clinical reasoning skills and appreciation of the socio-cultural context of care for shared decision-making. ● Responsibility ○ Explore the ethical and legal dimensions of care with increased use of AI. ○ Explore patients’ expectations and physicians’ responsibility for the care of patients as AI increasingly supports clinical decision-making.

In this study, residents expressed concern about their lack of agency in the current and future development and implementation of AI into their practice. They suggested that future AI development would be mainly in the hands of a profit-motivated private sector and well-resourced universities. Additionally, a variability in residents’ understanding of AI was noted during focus groups, with some discussing AI as involving static algorithms, along with the low number of residents with self-reported experience with AI. A paucity of education on AI and lack of opportunities to contribute to the development of AI technologies will limit the ability of physicians to contribute to the future of AI in healthcare. Foundational concepts of AI should be taught in required curricula with elective opportunities offered to deepen experience in AI when possible. Additionally, cross-departmental meetings and relationships (e.g., computer science and medicine) should be fostered at institutions where this is possible in order to create opportunities for collaboration for medical trainees. Educators have argued that learners should have opportunities to ‘co-develop, refine, validate, and spread AI’ ^7^; creating cross-departmental bridges during training may help to nurture a future where physicians are better equipped to contribute to development of technologies that will affect their own practice.

Residents cautioned that AI clinical decision support might obviate the need for physicians’ understanding of biomedical sciences, and that routine use of such technology might erode clinical reasoning that is supported by a contextual understanding of pathophysiology, a skill identified by residents as essential to the role of the physician. Furthermore, residents expressed concerns that AI might dispense clinical recommendations without a capacity to convey its reasoning to clinicians (i.e., black box phenomenon). Thus, as clinical practice becomes increasingly reliant on AI, educators must ensure that trainees’ clinical decision-making remains grounded in the clinical and biomedical sciences foundational to practice. This will prepare clinicians for effective clinical decision-making when AI-based decision support is unavailable or when a clinician’s thinking is at odds with guidance provided by AI-based support, particularly as the clinician may be unable to query the AI’s ‘thought’ process. In this way, assistance provided by AI can be presented in formal curricula as an additional source of data requiring interpretation and competence in probabilistic medicine and clinical reasoning, training for which may be lacking in many curricula ^26^. Recognition of biases that may be embedded within AI that may worsen health care disparities should be an extension of growing curricula on racism and structural inequities in healthcare ^27^.

Lastly and importantly, residents expressed that, despite the variety of assistive tools that AI will undoubtedly confer, physicians should remain responsible for shared decision-making with the patient - a recognition of the physician’s fiduciary responsibility for the overall care of the patient. Residents expressed that there should always be a physician involved in patient care even when AI is being used, and even provided examples of how they should be able to override certain AI-based decisions. These ideas underscore responsibility as a central element of the professional identity of physicians, a value that should continue to be emphasized in medical education given the changes that AI is bringing. Finally, the notion of critically grappling with recommendations from AI, which may sometimes be aligned with institutional initiatives for cost-containment, represents an opportunity to explore ethical reasoning in curricula. 

This study has certain limitations. The residents interviewed in this study were drawn from a single academic health center. Residents were sampled from several specialties, but did not include those from surgical specialties or dermatology, who may have had different perspectives regarding AI. The data collected is from 2018–2019 and reflects resident perception at that time. A variable understanding of the definition of AI amongst the residents was an interesting and unexpected finding; however, when discussing the impact of AI on healthcare, the residents mostly envisioned an assistive technology, which is an accurate reflection of current AI potential. Thus, this finding was not felt to invalidate the broader conclusions of this study. Because participation in focus groups was voluntary, this study may have selected for residents who had prior interest in or strong opinions regarding AI. Variability in the number of participants by specialty might have resulted in an over-emphasis of ideas from those specialties with greater representation; upon review of the data, however, themes presented were consistent across specialty groups.

Future research in this area might include investigations of resident clinical decision-making in the presence of AI support, baseline knowledge of AI among physicians or trainees, and the extent to which medical schools and residency programs are currently incorporating AI into curricula.

## Ethics and consent

This study was granted ethical approval by the Institutional Review Board of CUIMC (#AAAR7376; 4/12/2018). Written informed consent was obtained from each participant. Participation was voluntary and did not affect resident standing, and no compensation or incentive was provided to participants. Participants were informed that focus group data would be kept confidential and would not be shared with residency leadership.

## Data Availability

Figshare: Resident Physicians’ Perceptions of Artificial Intelligence and Implications for Medical Education: A Qualitative Study. https://doi.org/10.6084/m9.figshare.27640254.v1 ^19^ The project contains the following underlying data: Combined Focus Group Transcripts. Figshare: Resident Physicians’ Perceptions of Artificial Intelligence and Implications for Medical Education: A Qualitative Study. DOI: https://doi.org/10.6084/m9.figshare.27640080.v1 ^18^. This project contains the following extended data: Focus Group Guide. Data are available under the terms of the Creative Commons Attribution 4.0 International Licence (CC-BY 4.0)

## Software availability statement

This project utilized proprietary software (Dedoose). Qiqqa, a free and open-source alternative, is available as follows:

- Source Code available from: https://github.com/jimmejardine/qiqqa-open-source
- Archived software available from: http://github.com/jimmejardine/qiqqa-open-source/tree/master/Qiqqa-Software-Installer-Releases
- License: GNU General Public License v3.0
