# Supplementary material for: Resident Physicians’ Perceptions of Artificial Intelligence and Implications for Medical Education: A Qualitative Study
Source: MedEdPublish (2016). 2025 Jul 2;15:29. [Version 1] doi: 10.12688/mep.20833.1 (PMC12853013; doi:10.12688/mep.20833.1)
Supplement: Peer review history

# Reviewer report: Reviewer response for version 1

**DOI:** 10.21956/mep.22303.r45243

**Published:** 2026-01-28

**Authors:** Helena Filipe

**Affiliations:** 1 Independent Researcher, Lisbon, Portugal

Thank you for the opportunity to review Resident Physicians’ Perceptions of Artificial Intelligence and Implications for Medical Education: A Qualitative Study. This manuscript addresses a timely topic, examining how resident physicians view the emergence of artificial intelligence (AI) in healthcare and what their perspectives might suggest for the future of medical education. The study offers a valuable contribution by focusing on trainees, a group yet underrepresented in discussions about AI’s future impact, and by situating the work within the broader transformation of healthcare systems.

 The title accurately signals the study population and qualitative approach. A word of caution for the use of the term perceptions, as it may overstate participants’ familiarity with AI when many respondents acknowledge limited knowledge or firsthand experience. Similarly, the implications for medical education captured by the study should be framed in this context. The abstract is clear, well structured, and effectively communicates the study’s aims, methods, and key findings.

 The introduction establishes the relevance of AI to clinical care. The claim that there is “no consensus regarding how to introduce AI into medical education” would benefit from expanding on a broader and contemporary body of scholarship. Given that literature on AI in medical education has expanded rapidly, thoroughly situating the study within this discourse would strengthen the manuscript’s rationale.

 The methods are clearly described and adhere to established qualitative standards. Ethical procedures are appropriately detailed, and the study design is transparent. The manuscript could benefit from explicitly stating the primary research question and providing the focus group prompts, which would help readers better contextualize participants’ responses. The authors ground their study in constructing grounded learning theory without elaborating on a specific theoretical framework. Drawing on theories like the sociology of professions, technological change, or professional identity formation could further deepen the interpretive lens.

 The results are well described, and the thematic analysis is enriched by thoughtfully selected quotations. The themes, ranging from concerns about job security and loss of agency to optimism about AI as a collaborative partner capable of relieving administrative burden, illuminate how trainees imagine AI’s role in shaping future workflows. The depiction of a physician-patient-AI triad is particularly compelling. Explicit evidence-informed perspectives would add value to the manuscript to more robustly inform curricular design, given participants’ limited AI exposure.

 The discussion is systematic and forward-looking, effectively synthesizing themes and offering practical considerations. The accompanying figure and table provide clear and helpful summaries. Findings describe anticipated changes in clinical practice, suggest implications for medical education, and recommendations for curricular change as a result of a single-setting exploratory study findings.

 In conclusion, this is a well-written manuscript that adds a useful, future-oriented trainee perspective to the growing literature on AI in healthcare. Elaborating on its theoretical grounding, expanding its literature base, and framing its educational implications within the findings of a single-center study would further enrich the manuscript. 

 The paper makes a meaningful contribution to ongoing conversations about how physicians and the profession at large can navigate, shape, and critically engage with AI-driven change.

Have any limitations of the research been acknowledged?

Yes

Is the work clearly and accurately presented and does it cite the current literature?

Yes

If applicable, is the statistical analysis and its interpretation appropriate?

No

Are all the source data underlying the results available to ensure full reproducibility?

Yes

Is the study design appropriate and is the work technically sound?

Yes

Are the conclusions drawn adequately supported by the results?

Yes

Are sufficient details of methods and analysis provided to allow replication by others?

Yes

Reviewer Expertise:

ophthalmologist with special interest in ocular surface diseases, dry eye, cataract surgery

I confirm that I have read this submission and believe that I have an appropriate level of expertise to confirm that it is of an acceptable scientific standard.

# Reviewer report: Reviewer response for version 1

**DOI:** 10.21956/mep.22303.r44427

**Published:** 2025-12-25

**Authors:** Sanaa Saeed

**Affiliations:** 1 NFC Institute of Engineering and Technology Training, Multan, Pakistan

1. Is the work clearly and accurately presented and does it cite the current literature?

 ☑ Yes

 The article is well-organised, clearly written, and cites relevant literature throughout (references 1–27). It contextualises the study within existing research and highlights gaps that justify the investigation.

 ---

 2. Is the study design appropriate and is the work technically

 sound?

 ☑ Yes

 The use of a constructivist grounded theory (CGT) approach is appropriate for exploring perceptions. Focus groups across multiple specialties provide diverse perspectives. Ethical approval and consent procedures are documented. While there are limitations (single institution, voluntary participation), these are acknowledged.

 ---

 3. Are sufficient details of methods and analysis provided to allow replication by others?

 ☑ Yes

 The methodology is described in detail: recruitment strategy, focus group structure, coding process, use of Dedoose for data management, and achievement of saturation. Reporting aligns with qualitative research standards.

 ---

 4. If applicable, is the statistical analysis and its interpretation appropriate?

 ☑ Not applicable

 This is a qualitative study; no statistical analysis was performed. The interpretation of qualitative data appears appropriate.

 ---

 5. Are all the source data underlying the results available to ensure full reproducibility?

 ☑ Partly

 Tables summarising participant numbers and self-reported AI experience are included. However, full transcripts or anonymised excerpts are not provided, likely due to confidentiality. This limits complete reproducibility but is common in qualitative research. 

 ---

 6. Are the conclusions drawn adequately supported by the results?

 ☑ Yes

 Themes identified from coding are well-supported by participant quotes. Limitations (e.g., single site, specialty representation, timeframe) are discussed. Conclusions about implications for medical education are logically derived from findings.

Is the work clearly and accurately presented and does it cite the current literature?

Yes

If applicable, is the statistical analysis and its interpretation appropriate?

Not applicable

Are all the source data underlying the results available to ensure full reproducibility?

Partly

Is the study design appropriate and is the work technically sound?

Yes

Are the conclusions drawn adequately supported by the results?

Yes

Are sufficient details of methods and analysis provided to allow replication by others?

Yes

Reviewer Expertise:

NA

I confirm that I have read this submission and believe that I have an appropriate level of expertise to confirm that it is of an acceptable scientific standard, however I have significant reservations, as outlined above.

# Reviewer report: Reviewer response for version 1

**DOI:** 10.21956/mep.22303.r42390

**Published:** 2025-08-30

**Authors:** Anand Nayyar

**Affiliations:** 1 Duy Tan University, da nang, Vietnam

The Paper needs the following Major revisions and is subject for re-review, and after re-review, the final decision on the paper will be rendered:

 1. Add more information to the Introduction and elaborate it more with regard to some Background data at the start, background, scope, problem definition and other related technical insights matching to the main theme of the paper.

 2. Add Objectives of the paper in 2-4 points at end of introduction and then add Organization of the paper.

 3. Now after Introduction, add some Literature review to the paper, and elaborate every paper with regard to what was proposed, novelty aspect and what experimental results are observed and then add: Research Gaps in existing studies that led to the design of proposed work.

 4. Add Materials and Methods. ANd under materials, discuss the Dataset, Data Pre-Processing, Data Augmentation and under methods discuss the techniques used as base for the dataset analysis.

 5. Discussion to be more elaborative with regard to some problem focus.

 6. Add some real time case study oriented dicussion.

 7. Add future scope to the paper.

Have any limitations of the research been acknowledged?

No

Is the work clearly and accurately presented and does it cite the current literature?

Yes

If applicable, is the statistical analysis and its interpretation appropriate?

Partly

Are all the source data underlying the results available to ensure full reproducibility?

Partly

Is the study design appropriate and is the work technically sound?

Yes

Are the conclusions drawn adequately supported by the results?

Yes

Are sufficient details of methods and analysis provided to allow replication by others?

Yes

Reviewer Expertise:

IoT, AI, Healthcare Informatics, Cloud Computing, Drones.

I confirm that I have read this submission and believe that I have an appropriate level of expertise to confirm that it is of an acceptable scientific standard, however I have significant reservations, as outlined above.
